# Dyslipidemia and Cardiovascular Disease: A Series of Epidemiologic Studies in Japanese Populations

**DOI:** 10.2188/jea.JE20100060

**Published:** 2010-07-05

**Authors:** Tomonori Okamura

**Affiliations:** Department of Preventive Cardiology, National Cerebral and Cardiovascular Center, Suita, Osaka, Japan

**Keywords:** total cholesterol, low density lipoprotein cholesterol, high density lipoprotein cholesterol, coronary artery disease, stroke, cohort studies

## Abstract

Although the causal relationships of high serum levels of total cholesterol (TC) and low-density lipoprotein cholesterol (LDL-C) with coronary artery disease (CAD) are well established, there have been few community-based epidemiologic studies of these relations in Japan. Furthermore, even when analysis is restricted to ischemic stroke, the relationship between dyslipidemia and stroke is very weak. Accordingly, it is difficult to perform cohort studies of dyslipidemia and cardiovascular disease. A series of studies, such as the NIPPON DATA (National Integrated Project for Prospective Observation of Non-communicable Disease and Its Trends in the Aged) cohort study of a representative sample of Japanese, have greatly increased existing evidence. NIPPON DATA80 revealed a clear positive relationship between TC and CAD, and indicated that reverse causality between hypocholesterolemia and liver disease may increase all-cause mortality in hypocholesterolemic Japanese. NIPPON DATA90 showed that serum high-density lipoprotein cholesterol (HDL-C) was inversely associated with all-cause mortality, even when HDL-C was very high. NIPPON DATA80 revealed that low-normal levels of serum albumin and TC are associated with a decline in activity during old age, especially in women. The Suita study—a unique cohort study of urban residents—showed that LDL-C and non–HDL-C were equally accurate in predicting the incidence of myocardial infarction. Further research of this quality is needed to ascertain the public health burden of dyslipidemia in Japan.

## INTRODUCTION

The causal relationships between high serum levels of total cholesterol (TC) and low-density lipoprotein cholesterol (LDL-C) and coronary artery disease (CAD) are well established in various ethnic populations.^1^^–^^3^ In addition, serum high-density lipoprotein cholesterol (HDL-C) levels are known to be inversely associated with the risk of CAD. Therefore, in developed countries, serum cholesterol levels are the main target for lipid management in most guidelines developed to prevent atherosclerotic disease.^3^^–^^5^ The Health and Medical Service Law for the Elderly was enacted in Japan in 1982, and all Japanese residents aged 40 years or older have the opportunity to undergo screening for TC (changed to LDL-C in April 2008); those with dyslipidemia are provided with health services such as health education and guidance to prevent CAD.^6^

The evidence is not strong for a positive association between the risk of stroke and either high serum levels of TC or LDL-C, at least in the Japanese population. Indeed, an epidemiologic study in Japan was the first in the world to report that community residents with low serum total cholesterol were more likely to develop intraparenchymal hemorrhage.^7^ Some researchers hypothesized that this unexpected result was due to an interaction between low serum cholesterol and low serum albumin, which is an indicator of malnutrition; however, no epidemiologic study has addressed this topic.

CAD mortality is lower in Japan than in Western populations. In populations that have had low mean cholesterol for a period of many decades, as is the case in Japan, new epidemiologic studies are needed to determine the benefits of health services and lipid-lowering medications. The present series of studies were performed to add to the body of evidence on this topic.

## OUTLINE OF COHORT STUDIES

### NIPPON DATA

The cohort studies of the National Survey on Circulatory Disorders, Japan, are referred to as NIPPON DATA (National Integrated Project for Prospective Observation of Non-communicable Disease and Its Trends in the Aged)^8^; which included 2 cohort studies. The baseline surveys were performed in 1980 and 1990 (NIPPON DATA80 and NIPPON DATA90).^9^^,^^10^ A total of 10 546 community dwellers (4640 men and 5906 women) aged 30 years or older from 300 districts participated in the 1980 survey; 8384 community dwellers (3504 men and 4880 women) aged 30 years or older from 300 randomly selected districts participated in the 1990 survey.

The details of the baseline survey of these cohorts have been previously reported.^8^^–^^10^ Nonfasting blood samples were obtained and sera were separated. In NIPPON DATA80, serum total cholesterol was analyzed using the Liebermann–Burchard direct method; serum albumin was analyzed using the bromocresol-green method at 1 central laboratory (present name: Osaka Medical Center for Health Science and Promotion). In NIPPON DATA90, serum total cholesterol and triglyceride (TG) concentrations were measured enzymatically, and HDL-C was measured by the precipitation method using heparin-calcium at SRL, Inc. The precision and accuracy of the cholesterol measurements in the these laboratories have been certified by the CDC-NHLBI Lipid Standardization Program of the Centers for Disease Control and Prevention (CDC), Atlanta, Georgia.^11^

During the follow-up periods, the underlying causes of death in the National Vital Statistics were coded according to the 9th International Classification of Disease (ICD-9) for deaths occurring until the end of 1994, and according to the 10th International Classification of Disease (ICD-10) for deaths occurring from the beginning of 1995. The details of the classification used in these studies are described elsewhere.^8^^–^^10^ Permission to use National Vital Statistics was obtained from the Management and Coordination Agency, Government of Japan.

Information on activities of daily living (ADL) among the survivors of NIPPON DATA80 was collected by physicians and public health nurses at public health centers in 1994. Participants were asked about 5 basic ADL items (eating, dressing, bathing, toileting, and transferring–walking indoors), modified from Katz et al,^12^ and whether each of these activities could be accomplished without help, with partial help, or with full help. This survey was conducted via telephone interviews (30.5%), face-to-face interviews at home (43.5%), mail (9.0%), and other methods (17.0%). Impaired ADL was defined as a need for partial or full support to perform any of the 5 basic ADL items.

### Suita study

The Suita study, a cohort study of cardiovascular disease, was established in 1989 and included 12 200 Japanese urban residents of Suita City, Osaka. The participants were aged 30 to 79 years and were selected randomly from the municipal population registry. Of these, 6485 men and women had a baseline medical examination at the National Cardiovascular Center between September 1989 and February 1994. Blood samples were collected after the participants had fasted for at least 10 hours. The samples were centrifuged immediately and serum levels of TC, HDL-C, and TG were enzymatically measured. The details of the baseline survey have been reported elsewhere.^13^^–^^16^

The endpoints of this study were: (1) first myocardial infarction (MI) or stroke event, (2) death, (3) leaving Suita City, or (4) 31 December 2005. The first step in the survey of MI and stroke was monitoring the health status of all participants by means of clinic visits every 2 years, and by yearly questionnaires administered by mail or telephone. In the second step, in-hospital medical records of participants who were suspected of having had an MI or stroke were reviewed by registered hospital physicians or research physicians who were blinded to the baseline information. The criteria for definite and probable MI were defined according to the criteria of the MONICA (Monitoring Trends and Determinants of Cardiovascular Disease) project, which requires evidence from electrocardiography, cardiac enzymes, and/or autopsy.^17^ Stroke was defined according to the National Survey of Stroke criteria, ie, rapid onset of a constellation of neurological deficits lasting at least 24 hours or until death.^18^

## IMPORTANT FINDINGS

### Association of TC with cause-specific and all-cause mortality (NIPPON DATA80)^9^

No study has shown a positive relationship between hypercholesterolemia and all-cause mortality in the Japanese population. Therefore, 9216 participants of NIPPON DATA80 who had no missing data and no history of cardiovascular disease were followed until 1999.^9^ The mean follow-up period was 17.3 years, and the total number of person-years of follow-up was 159 293. During the follow-up period there were 1841 deaths (992 males and 849 females). Of these, 36% (*n* = 666) were due to cardiovascular disease, including 128 coronary heart disease deaths and 306 stroke deaths (intracerebral hemorrhage, *n* = 65; cerebral infarction, *n* = 174; others, *n* = 67).

**Figure 1.**
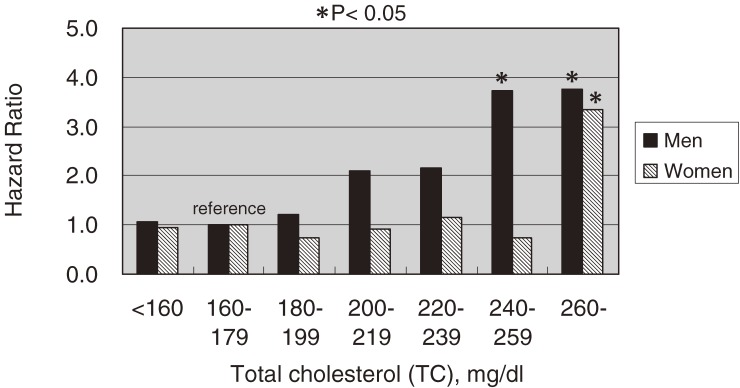
Multivariate-adjusted hazard ratios (HRs) for coronary artery disease mortality, grouped according to serum total cholesterol, after adjustment for age, serum albumin, body mass index, hypertension, diabetes, cigarette smoking, and alcohol intake. Black bars indicate HRs for men, and hatched bars indicate HRs for women. (*P < 0.05)

Figure 1 shows the relationship between TC and death due to coronary heart disease, and suggests that there is a positive graded relationship between these 2 parameters, especially in men. For men, the hazard ratio (HR) for the second-highest TC group (TC, 240–260 mg/dl) was 3.74 (95% CI, 1.44–9.76), while the HR for the highest TC group (TC, ≥260 mg/dl) was 3.77 (95% CI, 1.02–13.9). For women, although a graded relationship was not observed, the highest TC group had a significantly increased risk of death from coronary heart disease. Mortality from stroke was not associated with TC in either sex.

**Figure 2.**
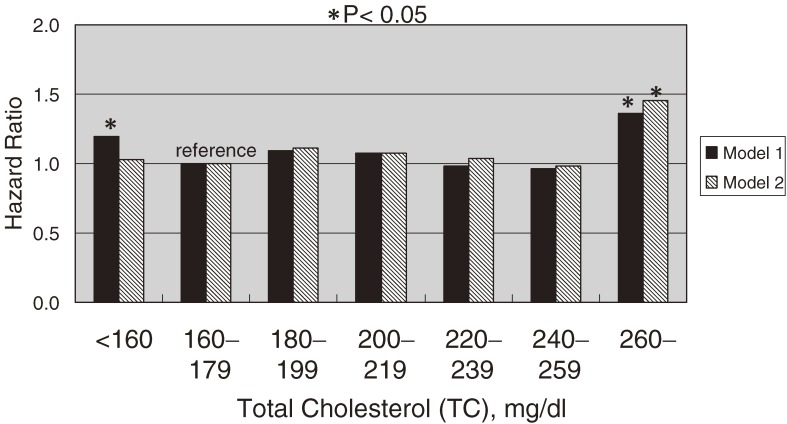
Multivariate-adjusted hazard ratios (HRs) for all-cause mortality, grouped according to serum total cholesterol, after adjustment for sex, age, serum albumin, body mass index, hypertension, diabetes, cigarette smoking, and alcohol intake. Black bars (model 1) indicate HRs for all-cause mortality among all participants. Hatched bars (model 2) indicate HRs for all-cause mortality after exclusion of deaths due to liver disease during the entire follow-up period and all-cause deaths within the first 5 years of follow-up. (*P < 0.05)

Concerning all-cause mortality (Figure 2), there was a positive association between TC and the risk of all-cause mortality in the group with the lowest TC (<160 mg/dl; HR, 1.19; 95% confidence interval [CI], 1.03–1.37) and the group with the highest TC (≥260 mg/dl; 1.36; 1.05–1.77). The lowest TC group also had an increased risk of liver disease (HR 3.03; 95% CI, 1.70–5.43). After excluding deaths due to liver disease during the entire follow-up period, and all-cause deaths within the first 5 years of follow-up, the increased HR in the lowest TC group disappeared (1.05; 0.89–1.24).

Most cohort studies in non-Western populations have failed to demonstrate a positive relationship between high serum TC and all-cause mortality. Serum TC levels were markedly lower among Japanese born before World War II, as compared with current levels. Even if some participants had elevated TC at the time of the baseline survey, the duration of this elevation from onset until the baseline measurement might have been short. The possibility that there is a “lag time” between exposure to high serum TC levels and the occurrence of coronary heart disease may explain the high cut-off value for TC needed to detect participants at high risk.

The prevalence of hepatitis C virus (HCV) infection in Japanese residents born before World War II has been estimated at 5% to 7%, a level considerably higher than that in Western countries.^19^ Because the majority of our study participants were born before World War II, the prevalence of HCV infection in NIPPON DATA80 would be expected to be relatively high. It has recently been found that a low serum cholesterol level in individuals with chronic HCV infection is a predictor of both liver fibrosis and liver cancer.^20^ Accordingly, hypocholesterolemia in Japan is associated with persistent HCV infection. Rather than being a primary cause of liver fibrosis, low serum TC may be a response to liver dysfunction caused by progressive fibrotic changes. We believe these findings partly explain the relationship between low TC and all-cause death in Japan. This interpretation is supported by a previous study which found that a history of blood transfusion was associated with hypocholesterolemia in a rural Japanese community.^21^

### HDL-C and all-cause mortality (NIPPON DATA90)^10^

**Figure 3.**
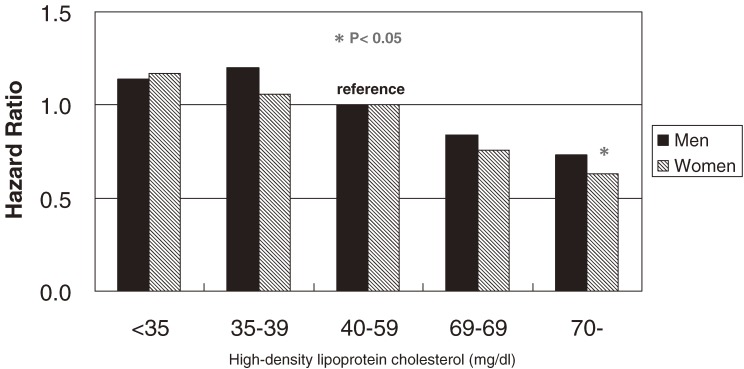
Multivariate-adjusted hazard ratios (HRs) for all-cause mortality, grouped according to serum high-density lipoprotein cholesterol (HDL-C), after adjustment for age, body mass index, triglyceride (log-transformed), non–HDL-C, hypertension, diabetes, cigarette smoking, and alcohol intake. Black bars indicate HRs for men, and hatched bars indicate HRs for women. (*P < 0.05)

In populations, such as Japanese, with HDL-C levels higher than those of Western populations, the beneficial effects of HDL-C on all-cause mortality may differ. A total of 7175 community-dwelling Japanese residents without a past history of cardiovascular disease in NIPPON DATA90 were followed for 9.6 years.^10^ During follow-up, there were 636 deaths. The multivariate-adjusted HR of HDL-C for all-cause or cause-specific mortality was calculated using a Cox proportional hazards model adjusted for other cardiovascular risk factors. All-cause mortality appeared to have an inverse graded relation with HDL-C (Figure 3). The HR for those with very high HDL-C (≥70 mg/dl), as compared with the reference group (40–59 mg/dl), was 0.73 (95% CI, 0.50–1.06) for men and 0.63 (0.41–0.94) for women. When analyzed as a continuous variable, serum HDL-C was significantly inversely associated with all-cause mortality. There was an inverse graded relation between cardiovascular mortality and HDL-C categories, but the association was nonsignificant.

There are several reasons for the higher serum HDL-C level in Japanese. As compared with whites, Japanese have a much lower body mass index and a higher prevalence of genetic cholesteryl ester transfer protein (CETP) deficiency.^22^ It is controversial as to whether very high levels of serum HDL-C are protective against coronary heart disease, as few epidemiologic studies have investigated the relationship between high HDL-C levels, or CETP deficiency, and CAD. The only prospective study of this association showed a low risk of CAD in Hawaiian Japanese with high HDL-C (≥60 mg/dl), irrespective of CETP deficiency.^23^ A large cross-sectional study of a southwest Japanese community, who underwent assessment for genetic CETP deficiency, also showed a low prevalence of coronary heart disease in participants with very high HDL-C (≥80 mg/dl).^24^ These studies suggest that a markedly increased level of serum HDL-C may be protective against CAD in Japanese.

### TC, albumin, and ADL (NIPPON DATA80)^25^

Decreased concentrations of serum albumin, a measure of nutritional and anti-inflammatory status, are associated with increased mortality.^26^ Low serum albumin also correlates with impaired ADL among community-dwelling individuals, impaired extremity function, and decreased skeletal muscle mass, especially among elderly individuals.^27^

The association of serum albumin and TC levels with an increased risk of subsequent mortality and/or future decline in ADL was also examined.^25^ The cohort consisted of 1844 Japanese individuals aged 60 to 74 years from NIPPON DATA80, who were followed until the first follow-up survey of ADL in 1994. The mean follow-up period was 12.4 years. After adjusting for age, TC, hypertension, diabetes, body mass index, cigarette smoking, and alcohol consumption, the multivariate odds ratios (OR) of impaired ADL was highest among women in the lowest albumin quartile (≤4.0 g/dl). The multivariate OR for the composite outcome of death or impaired ADL for the lowest albumin quartile, as compared with the highest quartile, was 1.56 (95% CI, 1.94–2.57) for men and 3.06 (1.89–4.95) for women.

**Figure 4.**
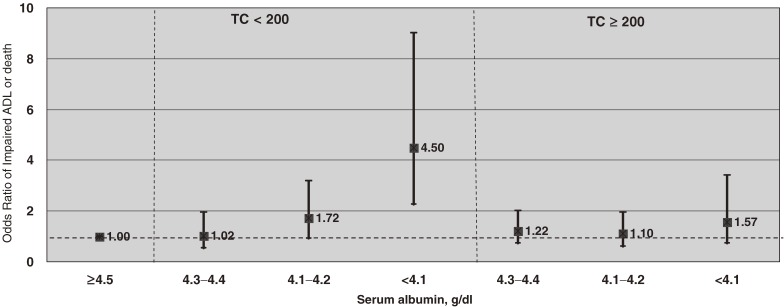
Multivariate odds ratios (ORs) and 95% confidence intervals (CIs) for the composite outcome of low activities of daily living (ADL) or death, according to quartile of serum albumin, after stratification at median (200 mg/dl) serum total cholesterol (TC), in women. The model was adjusted for age, TC, diabetes, hypertension, body mass index, cigarette smoking, and alcohol intake.

As shown in Figure 4, this relationship was more evident in participants with TC below the median: the multivariate OR for the lowest albumin quartile was 1.91 (95% CI, 0.88–4.15) in men and 4.50 (2.25–9.02) in women. When analyzed as a continuous variable, serum albumin was significantly and inversely associated with a composite outcome of death or impaired ADL in both men and women with TC below the median: the multivariate OR for each 0.1 g/dl increase in serum albumin was 0.88 (0.79–0.97) in men and 0.79 (0.72–0.87) in women; there was no significant association in those with TC above the median.

In a follow-up study of 937 US men and women aged 70 to 79 years, Reuben et al found that those with low albumin (≤3.8 g/dl) and low cholesterol (≤167 mg/dl) had the highest relative risks for 3- and 5-year mortality, and for the combined endpoint of 3-year mortality or decline in functional status.^27^ A decline in functional performance was 3 times more likely among Dutch men aged 55 to 85 years with serum albumin ≤4.3 g/dl and serum total cholesterol ≤200 mg/dl, than among those with higher levels, after 3 years of follow-up.^28^

In contrast, an inverse relationship between serum albumin and the incidence of coronary heart disease was observed among individuals with serum cholesterol levels ≥250 mg/dl in a 5-year follow-up study of 820 Dutch men aged 64 to 84 years.^29^ Interestingly, in the NIPPON DATA80 cohort, a combination of low albumin (≤4.3 g/dl) and above-average TC (≥185 mg/dl) was associated with excess mortality among participants aged 30 to 59 years.^30^ Thus, the interaction between serum albumin and serum cholesterol levels may be affected by the age of the surveyed population.

### LDC-C, Non–HDL-C, and the incidence of myocardial infarction (Suita study)^13^

Serum LDL-C levels are the main target for lipid management in the majority of guidelines promulgated by developed countries for preventing atherosclerotic disease.^3^^–^^5^ Some US cohort studies have suggested that non–high-density lipoprotein (non–HDL-C), which is calculated by subtracting HDL-C from TC, may be a better predictor of CAD.^31^ However, to our knowledge, only 1 population-based cohort study has directly compared the predictive value of these lipid markers for CAD in an Asian population.^32^

**Figure 5.**
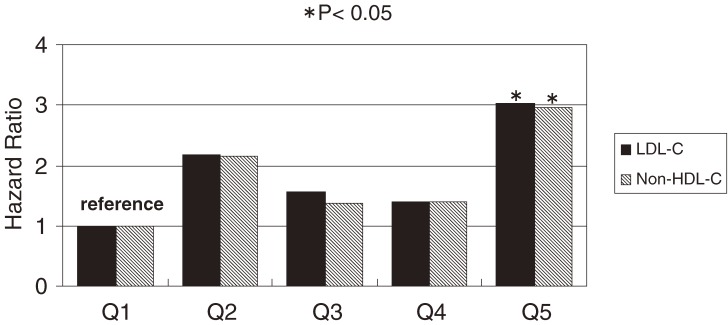
Multivariate-adjusted hazard ratios (HRs) for the incidence of myocardial infarction, grouped according to serum low-density lipoprotein cholesterol (LDL-C) or non–high-density lipoprotein cholesterol (non–HDL-C), after adjustment for sex, age, body mass index, hypertension, diabetes, HDL-C, cigarette smoking, and alcohol intake. Black bars indicate HRs of LDL-C; the hatched bars indicate HRs of non–HDL-C. (*P < 0.05) ‘Q’ indicates quintile. The cutoff points for LDL-C were Q1: <98, Q2: 98–117, Q3: 118–132, Q4: 133–150, Q5: ≥151 mg/dl for men, and Q1: <106, Q2: 106–124, Q3: 125–141, Q4: 142–163, Q5: ≥164 mg/dl for women. The cutoff points for non–HDL-C were Q1: <123, Q2: 124–142, Q3: 143–159, Q4: 160–179, Q5: ≥180 mg/dl for men, and Q1: <125, Q2: 125–142, Q3: 143–164, Q4: 165–188, Q5: ≥189 mg/dl for women.

A total of 4694 men and women aged 30 to 74 years without a history of cardiovascular disease or use of lipid-lowering medication underwent fasting blood collection and were followed for 11.9 years. Baseline LDL-C levels were estimated using the Friedewald formula, and the predictive value of LDL-C and non–HDL-C for MI and stroke were compared. During follow-up, there were 80 incident MIs and 139 stokes, comprising 23 intracerebral hemorrhages, 85 cerebral infarctions, and 31 strokes of another type. The HR for MI was highest (3.03; 95% CI, 1.32–6.96) among those in the top quintile of LDL-C (≥151 mg/dl in men and ≥164 mg/dl in women), when data from men and women were combined, as compared with the lowest quintile (<98 mg/dl in men and <106 mg/dl in women). The HR for MI was also highest (2.97; 95% CI, 1.26–6.97) for the top quintile of non–HDL-C (≥180 mg/dl in men and ≥189 mg/dl in women), as compared with the lowest quintile (<123 mg/dl in men and <143 mg/dl in women). Analysis of trends showed a significant positive relationship between MI incidence and serum LDL-C and non–HDL-C levels (both *P* = 0.02). A summary of these results is shown in Figure 5. However, there was no relationship between the incidence of any subtype of stroke and either LDL-C or non–HDL-C.

To determine the predictive values of LDL-C and non–HDL-C, the difference between the −2 ln[L] of a model including each lipid and the −2 ln[L] of a model adjusted for other variable was calculated. The χ^2^ values for LDL-C and non–HDL-C were almost identical: 5.71 for LDL-C (*P* = 0.02) and 5.49 for non–HDL-C (*P* = 0.02). Furthermore, the area under the curve (AUC) of the receiver-operating characteristics curves, based on predictive probability targeting for MI, was also estimated. The AUC of LDL-C and non–HDL-C were identical. Although the predictive value of these parameters is nearly identical, non–HDL-C should be recommended as a screening marker for primary prevention of CAD in the community, as it is less expensive and simpler to use.

### Conclusion

There have been few community-based epidemiologic studies of the relationship between dyslipidemia and cardiovascular disease in Japan. One reason for this is the low incidence of CAD, which is strongly associated with dyslipidemia.^33^ Most cardiovascular disease in Japan is stroke, and even if we restrict our analysis to ischemic stroke, the relation between dyslipidemia and ischemic stroke is very weak.^9^^,^^13^^,^^34^ This makes it difficult to perform epidemiologic studies of dyslipidemia, and it is thus a challenging task to produce articles of interest on this topic if we are restricted to the use of a Japanese dataset. Although the atherogenic effect of dyslipidemia has been established not only in epidemiologic studies, but also in many pathological and biological studies, further high-quality cohort studies are required to determine the public health burden of dyslipidemia in Japan.
